# Non-HLA angiotensin-type-1 receptor autoantibodies mediate the long-term loss of grafted neurons in Parkinson’s disease models

**DOI:** 10.1186/s13287-024-03751-y

**Published:** 2024-05-12

**Authors:** Ana I. Rodríguez-Pérez, Pablo Garrido-Gil, Maria García-Garrote, Ana Muñoz, Juan A. Parga, Jose Luis Labandeira-García, Jannette Rodríguez-Pallares

**Affiliations:** 1 Research Center for Molecular Medicine and Chronic Diseases (CiMUS), Health Research Institute of Santiago de Compostela (IDIS), University of Santiago de Compostela, Santiago de Compostela, 15782 Spain; 2 Networking Research Center on Neurodegenerative Diseases (CIBERNED), Madrid, Spain

**Keywords:** Angiotensin, AT1, Autoimmunity, Cell grafts, Cell transplantation, Microglia, Neurodegeneration, Parkinson, Rejection, Renin-angiotensin system

## Abstract

**Background:**

Clinical trials have provided evidence that transplants of dopaminergic precursors, which may be replaced by new in vitro stem cell sources, can integrate into the host tissue, and alleviate motor symptoms in Parkinson´s disease (PD). In some patients, deterioration of graft function occurred several months after observing a graft-derived functional improvement. Rejection of peripheral organs was initially related to HLA-specific antibodies. However, the role of non-HLA antibodies is now considered also relevant for rejection. Angiotensin-II type-1 receptor autoantibodies (AT1-AA) act as agonists of the AT1 receptors. AT1-AA are the non-HLA antibodies most widely associated with graft dysfunction or rejection after transplantation of different solid organs and hematopoietic stem cells. However, it is not known about the presence and possible functional effects of AT1-AA in dopaminergic grafts, and the effects of treatment with AT1 receptor blockers (ARBs) such as candesartan on graft survival.

**Methods:**

In a 6-hydroxydopamine PD rat model, we studied the short-term (10 days)- and long-term (3 months) effects of chronic treatment with the ARB candesartan on survival of grafted dopaminergic neurons and microglial graft infiltration, as well as the effects of dopaminergic denervation and grafting on serum and CSF AT1-AA levels. The expression of AT1 receptors in grafted neurons was determined by laser capture microdissection.

**Results:**

At the early period post-grafting, the number of grafted dopaminergic neurons that survived was not significantly different between treated and untreated hosts (i.e., control rats and rats treated with candesartan), probably because, just after grafting, other deleterious factors are predominant for dopaminergic cell death, such as mechanical trauma, lack of growth factors/nutrients and ischemia. However, several months post-grafting, we observed a significantly higher number of surviving dopaminergic neurons and a higher density of striatal dopaminergic terminals in the candesartan-treated group. For several months, grafted rats showed blood and cerebrospinal fluid levels of AT1-AA higher than normal controls, and also higher AT1-AA levels than non-grafted parkinsonian rats.

**Conclusions:**

The results suggest the use of ARBs such as candesartan in PD patients, particularly before and after dopaminergic grafts, and the need to monitor AT1-AA levels in PD patients, particularly in those candidates for dopaminergic grafting.

**Supplementary Information:**

The online version contains supplementary material available at 10.1186/s13287-024-03751-y.

## Background

Clinical trials have provided evidence that transplants of fetal midbrain-derived dopaminergic precursors can integrate into the host tissue and alleviate motor symptoms [1] in Parkinson’s disease (PD) patients. However, these studies have also revealed several questions that must be addressed before widespread this therapy [2]. A major limitation is the poor survival of grafted dopaminergic cells [3]. Previous studies have shown that only about 10% of fetal dopaminergic precursors survive following transplantation, or even less when dopaminergic neurons derived from embryonic stem cells (ESCs) or induced pluripotent stem cells (iPSCs) are used [3–6]. Access to large amounts of cells from new sources may be easier, but the initial volume of cells implanted in the brain must be within a limit, and the survival of transplanted cells must be improved. The events preceding the tissue implantation and during the first-week post-grafting, such as hypoxia and limited availability of nutrients and trophic factors, as well as the host immune response, have been suggested to be responsible for the early massive cell death [5, 7, 8]. However, transplantation of dopaminergic neuroblasts in PD patients revealed that a deterioration of graft function and possible loss of dopaminergic neurons may also occur several months after observing a graft-derived functional improvement [9, 10]. This was related to no immunosuppression or withdrawal of immunosuppression [11] leading to some degree of graft rejection [9, 10].

Rejection of peripheral organs was initially related to HLA-specific antibodies. However, the role of non-HLA antibodies is now considered also relevant for rejection [12, 13]. Angiotensin II type 1 (AT1) autoantibodies (AT1-AA), which activate AT1 receptors, have been the non-HLA antibodies most widely associated with graft dysfunction or rejection [14, 15]. This has been particularly studied in kidney transplants, where AT1-AA were identified for the first time by Dragun et al. in patients with negative donor-specific HLA antibodies, being the AT1-AA effects inhibited by treatment with the AT1 receptor blocker (ARB) losartan [16]. Then, different studies have shown that both previously formed, and *de novo* formed (grafting-derived) AT1-AA are associated with negative transplant outcomes, a high risk of antibody-mediated inflammation, and a decrease in graft function several months after transplantation, or rejection [14, 15, 17]. Although most studies on AT1-AA have been performed in kidney transplants, more recent studies have observed similar effects in heart, lung, liver, and hematopoietic stem cell transplantation [18–21]. It is well known that AT1 receptors are expressed in most tissues including vascular endothelial cells, inflammatory cells, kidney, heart, lung, liver, and brain cells [22]. The peptide angiotensin II (Ang II), via AT1 receptors, is one of the most important inducers of inflammation and oxidative stress in different tissues [23–25] and plays a major role in the pathogenesis of several degenerative diseases [26, 27].

In the brain, and particularly in the nigrostriatal system, the presence of AT1 and other angiotensin receptors in dopaminergic neurons and glial cells has been shown [28–30] and AT1 overactivity induces the progression of neuroinflammation and dopaminergic degeneration in different experimental models [31–33]. Consistent with this, recent studies in humans have identified high levels of AT1 gene expression as a marker for the most vulnerable dopaminergic neurons [34] and that chronic treatment with ARBs reduces the risk of PD development [35, 36]. Interestingly, we have observed increased expression of serum and cerebrospinal fluid (CSF) AT1-AA in PD patients and PD experimental models. In experimental models, AT1-AA increased the progression of dopaminergic degeneration, which was inhibited by ARBs [37]. However, it is not known about the presence and possible functional effects of AT1-AA in dopaminergic grafts. In a rat model of PD, we studied the effects of ventral mesencephalic grafts on serum and CSF AT1-AA and short- and long-term effects of the ARB candesartan on survival of grafted dopaminergic neurons and microglial graft infiltration.

## Materials and methods

### Experimental design

**Fig. 1:**
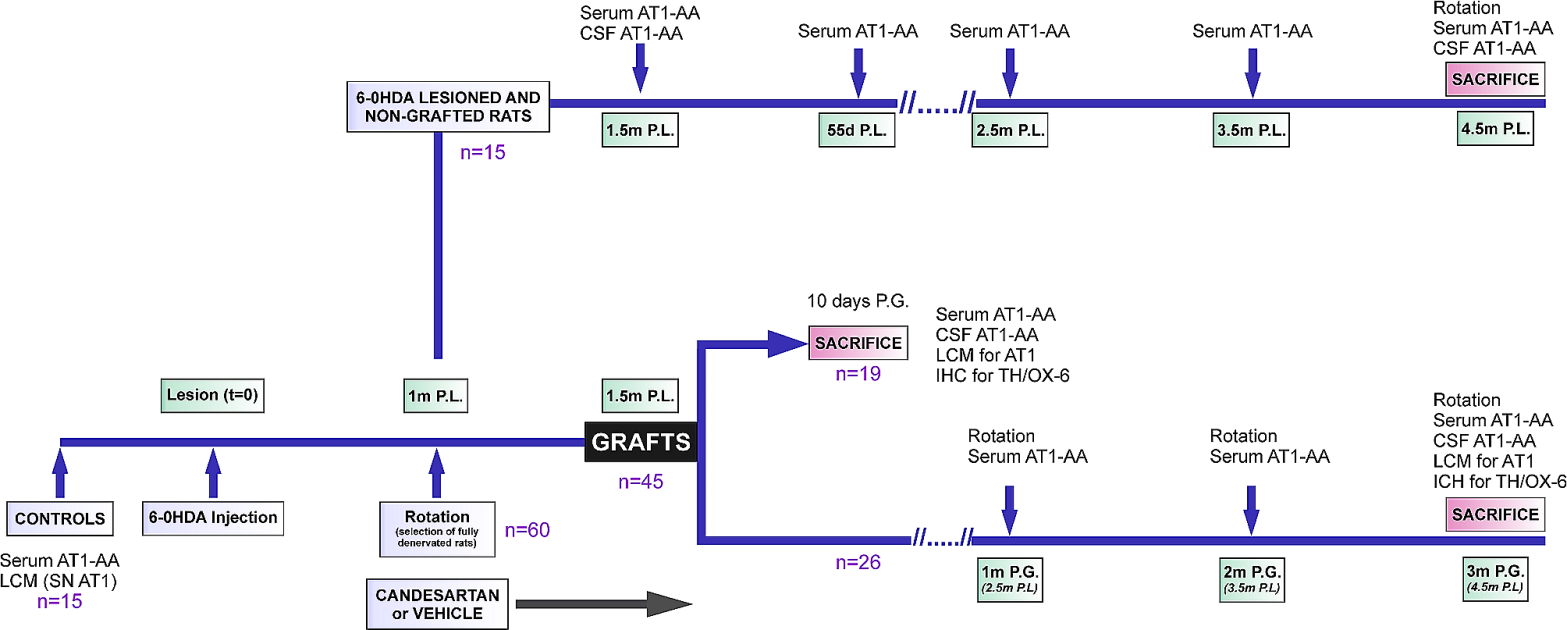
Experimental design. Time-course of experiments (0-4.5 months post-lesion) in the different groups of rats: rats lesioned with 6-OHDA, 6-OHDA lesioned rats and then grafted for a short period (short-term grafts; 10 days), and 6-OHDA lesioned rats grafted for 3 months (long-term grafts). Abbreviations: AT1-AA, angiotensin–type-1 receptor autoantibodies; CSF, cerebrospinal fluid; IHC, immunohistochemistry; LCM, laser capture microdissection; M, months; OX-6, microglial marker; P.L., post-lesion; P.G., post-grafting; SN, substantia nigra; TH, tyrosine hydroxylase

Adult female Sprague–Dawley rats (8 weeks old, weighing ∼ 250 g at the beginning of the study) were used. Female rats were used because the important increase in body weight of male rats in long-term experiments, such as those analyzing long-term grafts, may affect motor behavioral tests. Rats were housed at constant room temperature (21–22 °C) and 12-h light/dark cycle with access to food and water *ad libitum*. All experiments were carried out under the Spanish (RD53/2013) and European Communities Council Directive (2010/63/EU), in compliance with ARRIVE guidelines, and were approved by the corresponding committees at the University of Santiago de Compostela and Galician Government (15,012/2021/012). For surgical procedures, rats were deeply anesthetized with ketamine (75 mg kg^− 1^)/ xylazine (10 mg kg^− 1^) and maintained on a heated pad to facilitate maintenance of body temperature. First (Fig. 1), control rats (*n* = 15) were used to measure serum and cerebrospinal fluid (CSF) levels of AT1-AA, and levels of AT1 receptor mRNA expression in the ventral and dorsal region of the rat substantia nigra (SN). A second group of rats were subjected to maximal unilateral dopaminergic denervation with 6-hydroxydopamine (6-OHDA). One month after the 6-OHDA injections (4 weeks post-lesion, p.l.), rats with maximal lesions (dopaminergic depletion > 90%) were selected in a rotometer (*n* = 60). Two weeks later (1.5 months p.l.), serum and CSF (*n* = 15) samples were obtained from rats showing behavioral criteria for maximal dopaminergic denervation for the determination of AT1-AA, and other maximally lesioned rats were used for transplantation (*n* = 45) and organized into two series of experiments (Fig. 1). A first series of experiments was undertaken to investigate short-term grafts (i.e., 10-day grafts). Rats (*n* = 19) were transplanted with dopaminergic precursors (500,000 cells) derived from fetal (13 days of gestation, E13) ventral mesencephalon (VM) and killed 10 days post-grafting (p.g.; 55 days p.l.). Levels of AT1-AA were measured in serum and CSF samples after the sacrifice (*n* = 5). Levels of AT1 receptor mRNA were determined in a subgroup of grafted and not perfused rats (*n* = 5), and an additional subgroup of rats grafted from the same VM cell suspension (*n* = 9) were treated with the ARB candesartan (VM + candesartan) or vehicle (VM; see below), perfused before sacrifice and used for immunohistochemistry (IHC) to study effects of candesartan on the dopaminergic neuron survival and microglial response. The rats were treated with candesartan cilexetil (1 mg/kg/day; AstraZeneca, Madrid, Spain). Candesartan is an ARB that can cross the blood-brain barrier (BBB), and low doses of candesartan do not induce significant effects on blood pressure and inhibit the effects of Ang II in the CNS [38–40]. We administered candesartan orally in boluses mixed with Nocilla® hazelnut cream spread (Idilia Foods, Valencia, Spain) [41] from 15 days before transplantation until they were killed 10 days p.g. Control grafted rats (i.e., untreated grafted rats) received similar boluses without the drug (vehicle). Blood pressure was measured as in our previous studies [42, 43], using a non-invasive pressure system MRBP (IITC Life Science, California, U.S.A.), which measures blood pressure at the tail of the rats. Before the definitive measurement, the animals were preconditioned in the system (2 previous measurements) to avoid the increase of blood pressure eventually caused by stress. All measurements were carried out maintaining the animals at a temperature between 35 and 37 °C, which allows caudal artery dilatation, and promotes higher accuracy and precision in the measurements. There were no significant differences between the blood pressure values of the control and candesartan-treated rats (105.143 mmHg ± 3.87 and 94.43 mmHg ± 3.16, respectively; Mean ± SEM).

In a second series of experiments, we studied long-term grafts. Rats were transplanted with dopaminergic precursors (500,000 cells) derived from fetal VM and sacrificed 3 months p.g. Before the sacrifice (1, 2, and 3 months p.g.), rats were tested in the rotometer and levels of AT1-AA were measured in serum samples, and AT1-AA were also determined in the CSF after the sacrifice (*n* = 7). Levels of AT1 receptor mRNA were determined (3 months p.g.) in a subgroup of grafted and not perfused rats (*n* = 5); an additional subgroup of rats grafted from the same cell suspension (*n* = 14) were treated with the ARB candesartan (VM + candesartan) or vehicle (VM) as above, perfused before sacrifice and used for IHC to study effects of candesartan on dopaminergic neuron survival and the microglial response. In the grafts (10 days or 3 months p.g.) prepared for IHC we used tyrosine hydroxylase (TH) to identify and quantify dopaminergic neurons and terminals, and OX-6 IHC to identify and quantify so-called activated microglia infiltrating the grafts. We also measured the size of the grafts and the reinnervation area. Finally, part of the 6-OHDA-lesioned rats (*n* = 15) was not grafted and used for comparison (rotation, serum, and CSF AT1-AA) with grafted rats at similar time points (Fig. 1).

### 6-OHDA lesion and transplantation surgery

Rats were deeply anesthetized as described above (i.e. with ketamine /xylazine, 75 mg/ kg and 10 mg/ kg, respectively). Unilateral lesions of the dopaminergic system were performed by injection into the right, medial forebrain bundle of 12 µg of 6-OHDA HBr (in 4 µl of sterile saline containing 0.2% ascorbic acid; Sigma-Aldrich, Merck, Madrid, Spain). The stereotaxic coordinates were: 3.7 mm posterior to the bregma, 1.6 mm lateral to midline, and 8.8 mm ventral to the skull, with the tooth bar set at -3.3 mm [44]. Mesencephalic cell suspensions were prepared as follows: The pieces of VM were dissected out and incubated in 0.1% trypsin (Sigma-Aldrich), 0.05% DNase (Sigma-Aldrich), and DMEM (Gibco, Thermo Fisher Scientific, Waltham, MA, USA) for 20 min at 37 °C. The tissue was then rinsed in DNase/DMEM and mechanically dissociated to obtain a cell suspension. A total of 3 µl of cell suspension containing 500,000 VM cells was slowly administered to each rat into the right striatum at a single injection site (anterior to bregma = 0.6 mm, lateral to midline = 2.7 mm, ventral to the dura = 4.5 mm, with the tooth bar set at 0 mm) [44, 45]. As it is known that the viability of the dopaminergic cells in the VM suspension decreases over time, the grafts were performed in two sessions using new cell suspensions for the VM and VM + candesartan-treated groups, which were compared with the corresponding controls of rats grafted with the same VM suspension. In addition, within each grafting session, control (VM) and treated (VM + candesartan) rats were grafted alternately to minimize the time-dependent decline of dopaminergic cell viability.

### Behavioral testing of lesion efficacy and graft survival

The efficacy of the lesion and graft survival were evaluated one month after the lesion and 1, 2, or 3 months after grafting in the rats included within the long-term survival experiments (i.e., 3 months p.g.) by the rotometer test [46]. The efficacy of the lesion and graft survival were also confirmed by subsequent TH (as a dopaminergic marker) IHC of the SN and striatum at the end of the experiments (see below). Drug-induced rotation was tested in a bank of eight automated rotometer bowls (Rota-count 8, Columbus Instruments, Columbus, OH, USA). Turning behavior was monitored for 90 min after injection of D-amphetamine (5 mg/kg in saline; intraperitoneally; Sigma-Aldrich). Rats showing at least 7 net full turns towards the lesioned side were identified as maximally lesioned animals and were included in the study.

### Immunolabelling and quantitative histological analysis

Animals were killed with an overdose of ketamine/xylazine anesthesia, and immediately perfused with cold 4% paraformaldehyde in 0.1 M phosphate buffer pH 7.4. Brains were dissected out, cryoprotected in the same buffer containing 30% sucrose, and cut with a microtome into 40 μm-thick coronal sections. Series of free-floating sections were preincubated for 1 h in a blocking solution containing 10% normal serum with 0.25% Triton™ X-100 (Sigma-Aldrich) in 0.02 M potassium phosphate-buffered saline containing 1% bovine serum albumin (KPBS-BSA) at room temperature. The sections were then incubated overnight at 4 °C with the corresponding primary antibody: mouse monoclonal antibody against TH (1:10000; T2928; Sigma-Aldrich) or mouse monoclonal antibody against OX-6 (as a marker of activated microglia; 1:500; MCA46G; BioRad, Hercules, CA, USA). The sections were subsequently incubated, first for 1 h with the corresponding biotinylated secondary antibody (horse anti-mouse; 1:200; Ba-2001; Vector Laboratories, Newark, CA, USA) and then for 90 min with an avidin–biotin–peroxidase complex (ABC complex; 1:150; Vector Laboratories). The labelling was visualized using 3,3´-diaminobenzidine (DAB; Sigma-Aldrich) for TH or DAB in a nickel sulphate solution for OX-6 [44, 45].

Quantification of TH-immunoreactive (-ir) neurons or OX-6-ir cells in grafts was carried out in every fourth section to cover the entire graft from the rostral tip to the caudal end. Sampling was carried out with the Computer Assisted Stereological Toolbox (CASTGrid system; Olympus, Ballerup, Denmark) and the observer was blind. The total number of TH-positive neurons or OX-6-ir cells infiltrating the grafts were calculated according to the optical fractionator formula [47]. A counting frame (1800 µm^2^) was placed at random on the first counting area and systematically moved through all counting areas until the entire delineated area was sampled. Cell profiles were observed with a 100x oil objective (Numerical Aperture, NA 1.4). The graft volume was estimated according to Cavalieri’s method [48]. The striatal graft-derived reinnervation area (TH-ir area surrounding the graft) was measured using the CASTGrid system. At least three sections throughout the grafted striatum of each rat were measured and expressed in mm^2^. The density of the TH-ir fibers in the graft-derived reinnervation area was estimated as the optical density of striatal TH-ir with the aid of NIH-Image 1.55 image analysis software. At least three sections throughout the graft of each animal were measured, and for each section optical density was corrected by subtraction of background as observed in the corpus callosum.

### Laser capture microdissection of grafted dopaminergic neurons

Laser capture microdissection (LCM) of dopaminergic neurons from grafts and SN (dorsal or ventral tiers) was performed as previously described [49]. Briefly, rats were killed by an overdose of ketamine/xylazine anesthesia, immediately decapitated, and their brains were then rapidly removed, embedded in Tissue-Tek® O.C.T. (Sakura Fineteck Inc, Torrance, CA, USA), frozen in liquid nitrogen, and stored at − 80 °C. Serial coronal sections (20-µm thick) containing the graft and the SN were cut on a cryotome, mounted on RNase-free glass slides, and stored at − 80 °C in sterile Falcon tubes containing silica gel until further processing. Then, series of sections through the grafts or the SN were processed for a shortened immunofluorescence method with an antibody against TH (1:100) as a dopaminergic marker. LCM was performed using a PALM MicroBeam (Zeiss, Oberkochen, Germany) system controlled by PALM Robo software. Dopaminergic neurons from the dorsal or ventral SN of intact rats (*n* = 5) were used as controls. Dorsal and ventral tiers of the SN were delimitated according to [50]. Dopaminergic neurons were identified under fluorescence microscopy at 40x magnification. Cell pools (1000 dopaminergic neurons per animal; *n* = 5) were microdissected using the Palm software, and then cut and catapulted by laser pulses into an adhesive microtube cap (Zeiss). Neuronal pools were rapidly lysed for 30 min at RT in lysis buffer (RNeasy Microkit, Qiagen, Hilden, Germany) and stored at -80 ºC until RNA extraction.

### RNA extraction and real-time quantitative polymerase chain reaction

Total RNA extraction was performed using the RNeasy Micro kit (Qiagen) according to the manufacturer’s instructions. All RNA obtained for each sample was reversed transcribed (RT) to complementary DNA (cDNA) using nucleoside triphosphate containing deoxyribose, random primers and Moloney murine leukaemia virus (M-MLV; Invitrogen, Thermo Fisher Scientific) reverse transcriptase.

Subsequently, the real-time quantitative RT-PCR analysis was performed with a QuantStudio 3 platform (Applied Biosystems, Foster City, CA, USA), the EvaGreen qPCR MasterMix (Applied Biological Materials Inc., Vancouver, Canada), and the following primer sequences were used to examine the relative levels for AT1 receptors (accession number: NM_030985.4; forward 5’- TTCAACCTCTACGCCAGTGTG-3’ and reverse 5’-GCCAAGCCAGCCATCAGC-3’), and β-actin (accession number:

NM_031144.3; forward 5’- TCGTGCGTGACATTAAAGAG-3’ and reverse 5’- TGCCACAGGATTCCATACC-3’) was used as a housekeeping gene and was amplified in parallel with the AT1 gene. We used the comparative cycle threshold values (cycle threshold (Ct)) method (2 − ^ΔΔCt^) to examine the relative messenger RNA (mRNA) expression. The PCR products were loaded into a 2% agarose gel with Syber Safe (Invitrogen), separated by electrophoresis, and visualized with an UV detection system (Molecular Imager Chemidoc XRS System, BioRad).

### Extraction of blood and CSF from rats

Repeated blood collection at different times was carried out in the lateral tail vein (including samples before the 6-OHDA lesions for controls, and before grafting for 6-OHDA-lesioned samples). After decapitation (see above), blood was immediately extracted by decantation. Then, serum was obtained by centrifugation at 2000 g for 10 min. Then, serum aliquots were stored at − 80 °C until processed. CSF was collected as previously described by Pegg et al. [51]. Briefly, rats were placed, under deep anaesthesia, in a stereotaxic frame with the head flexed downward at 45º. Muscle layers were separated along the midline to expose the duramater. Then, a 30G needle attached to a 1 ml syringe was inserted from the caudal end of the incision and at a 30º angle to the dura, and CSF was aspirated by pulling back the plunger of the syringe. CSF was rapidly stored at − 80 °C until processed for quantification of autoantibodies.

### Serum and CSF AT1-AA determination

Serum and CSF AT1-AA levels were measured using a specific solid-phase, sandwich enzyme-linked immunosorbent assay (ELISA) for the quantitative determination of these autoantibodies (Catalog Number 12,000; Cell Trend; Luckenwalde, Germany). The manufacturer’s instructions were followed, with a light modification as previously described [52] consisting of the use of an HRP-conjugated goat anti-rat (1:2500, ab97057, Abcam, Cambridge, UK) as a secondary antibody. Immunoreactivity was detected with an Immun-Star HRP Chemiluminescent Kit (170–5044, Bio-Rad) and luminescence was measured using an Infinite M200 multiwell plate reader (TECAN, Männedorf, Switzerland).

### Statistical analysis

All statistical analyses were performed using SigmaPlot 11.0 (Systat Software, Inc., CA, U.S.A.). Normality of populations and homogeneity of variances were tested before each analysis of variance by using the Kolmogorov–Smirnov test. If the dataset passed the normality test and homogeneity of variance, parametric tests were used: Student’s t-test for two-group comparisons and one-way analysis of variance (ANOVA) followed by the corresponding post-hoc tests, for multiple comparisons. For nonparametric data, two group comparisons were carried out by the Mann–Whitney rank sum test, and multiple comparisons were studied by using the Kruskal–Wallis one-way ANOVA on ranks test followed by corresponding post-hoc tests. All data were expressed as means ± standard error of the mean (SEM). Differences were considered statistically significant at *P* < 0.05. GraphPad Prism 8 software (GraphPad; Inc., San Diego, CA, USA) was used to create scatter dot plot graphs.

## Results

### Short-term effects of treatment with candesartan on graft dopaminergic neurons and microglial infiltration. Expression of neuronal AT1 receptors and AT1-AA

**Fig. 2:**
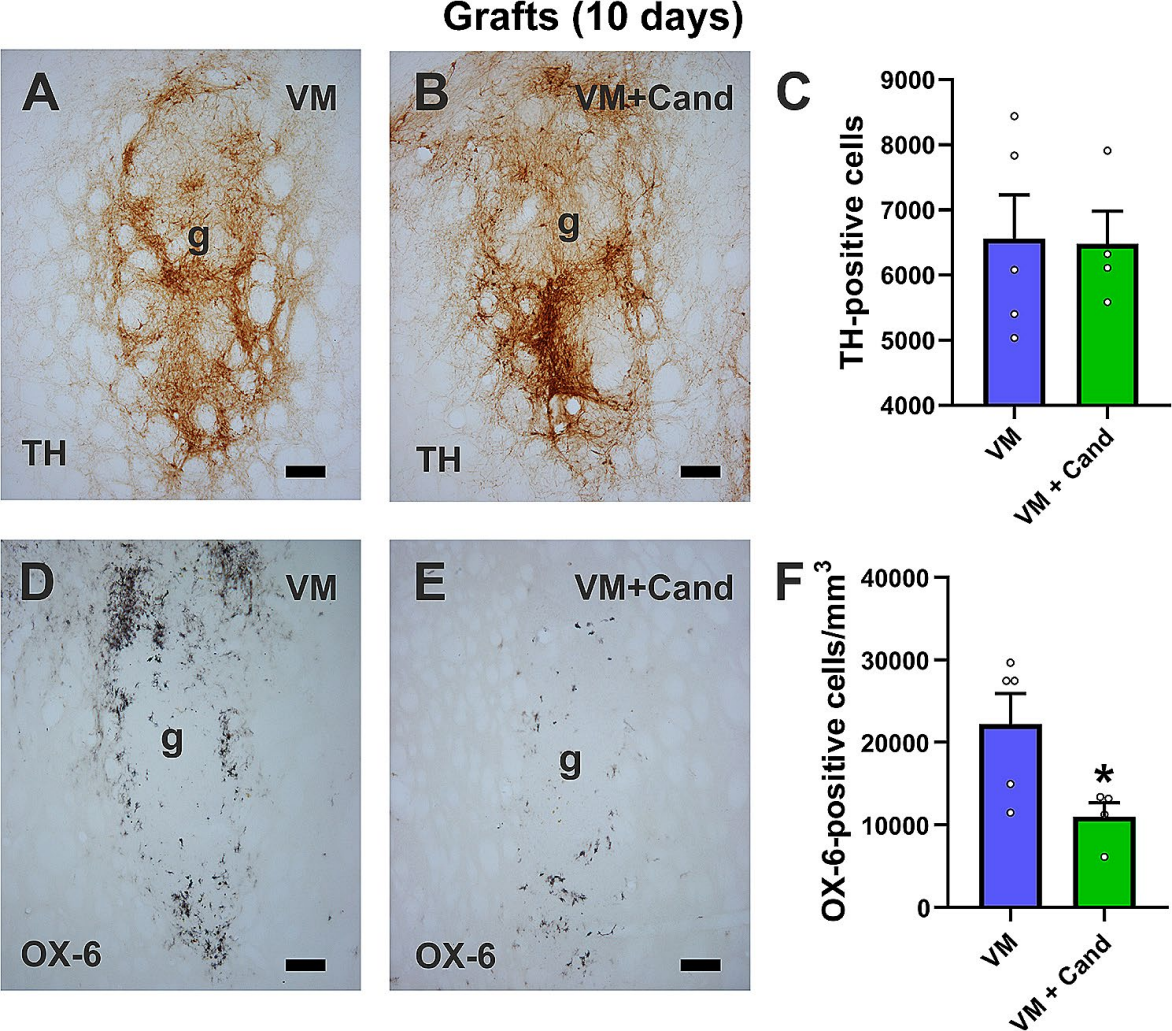
Short-term (i.e., 10 days post-grafting.) histological analysis of intrastriatal grafts (g). **A, B** Immunoreactivity (-ir) against TH (dopaminergic marker) and **D, E** OX-6 (microglial marker) in rats grafted with VM cell suspensions and treated with vehicle (i.e., control; **A, D**) or treated with the AT1 antagonist candesartan (i.e., VM + cand; **B**, **E**). **C** The number of TH-ir cells was similar in untreated and candesartan-treated animals. However, **F** the number of microglial OX-6 positive cells was significantly lower in the candesartan-treated group than in the untreated and grafted group. Data represent mean ± SEM. *p < 0.05 relative to untreated grafted rats (Student’s t-test). Scale bar = 75 μm. Abbreviations: SEM, standard error of the mean; TH, tyrosine hydroxylase; VM, ventral mesencephalon

To study the effect of AT1 receptor blockade on short-term dopaminergic cell survival, control grafts (i.e., untreated VM-grafted rats) and VM-grafted rats treated with candesartan (i.e., VM + candesartan) were sacrificed 10 days p.g. All grafted rats met behavioral criteria for maximal dopaminergic denervation. One month after the 6-OHDA lesion, the lesion induced a strong rotational asymmetry and amphetamine-induced ipsilateral turning (i.e., towards the denervated side) in both lesioned rats of the group to be grafted only with VM (1064 ± 109.4 turns) and the group to be grafted with WM and treated with candesartan (1047.8 ± 48.8 turns). IHC analysis showed the presence of many TH-positive cells in VM-grafted rats (i.e., untreated grafts). Most cells appeared scattered and large numbers of TH-ir fibers were observed within the graft (Fig. 2A). The number and distribution of TH-positive cells in grafted animals treated with candesartan was as observed in VM-grafted rats (Fig. 2B, C). Similarly, the average graft volume in the VM-grafted group was not significantly different from that observed in the grafted rats treated with candesartan (0.465 ± 0.076 mm^3^ and 0.327 ± 0.034 mm^3^, respectively).

Interestingly, the number of OX6-ir cells (i.e., the so-called activated microglial cells) infiltrating the grafts was significantly lower in VM + candesartan-grafted rats compared to untreated grafted animals (i.e., VM control grafts), which doubled in number the microglia infiltrating grafts in candesartan-treated rats (Fig. 2D-F). No graft-induced rotational changes can be expected at 10 days p.g., as a few weeks are necessary for the detection of graft functional effects.

**Fig. 3:**
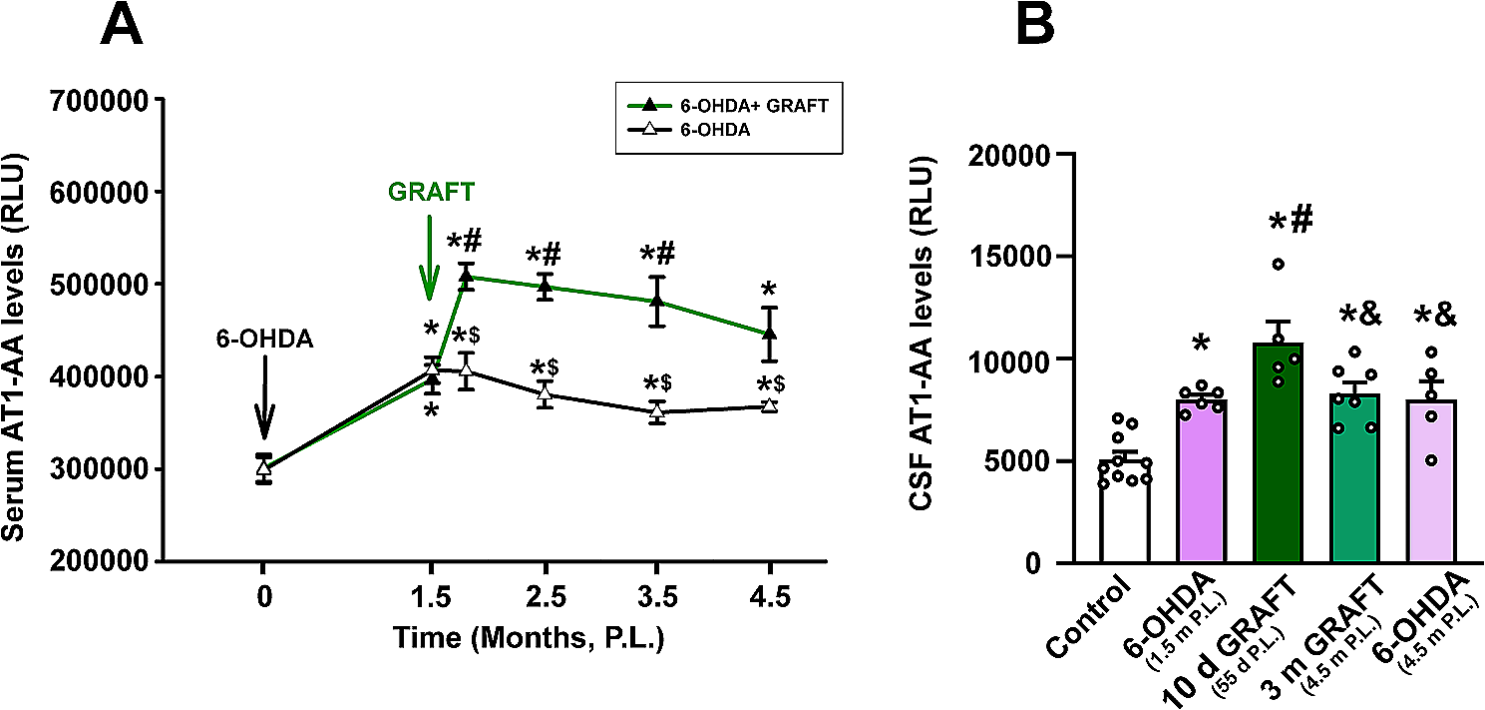
Serum and CSF levels of AT1-AA. **A** In grafted rats, serum levels of AT1-AA were significantly higher than in controls, and in non-grafted 6-OHDA-lesioned rats. **B** In the CSF, short-term grafts (10 days) showed levels of AT1-AA higher than controls and 6-OHDA-lesioned non-grafted rats, and long-term grafts showed levels of AT1-AA higher than control rats, and lower than in short-term grafts. Data are given as means ± SEM. * *p* < 0 0.05 compared to the control group; ^#^*p* < 0.05 compared to 6-OHDA (1.5-month P.L. group); ^$^*p* < 0.05 compared to the matched-grafted group; ^&^*p* < 0.05 compared to 10 days grafted animals group. One-way ANOVA followed by Holm Sidak as post-hoc test. Comparisons among 6-OHDA and matched grafted groups were carried out using Student t-tests (for 0, 1.5, 2.5 months P.L.) and Mann–Whitney rank sum test (for 55 days, and 3.5 and 4.5 months P.L.). Abbreviations: 6-OHDA, 6-hydroxydopamine; ANOVA, one-way analysis of variance; AT1-AA, autoantibodies for AT1 receptors; CSF, cerebrospinal fluid; m, months; P.L., post-lesion; RLU, relative luminescence units; SEM, standard error of the mean

Consistent with our recent studies in PD models and patients [37] 6-OHDA-lesioned rats showed pre-grafting levels of AT1-AA significantly higher than control healthy rats, both in serum and in CSF (Fig. 3). Interestingly, we observed an additional significant increase in serum levels of AT1-AA that continued higher than in non-grafted lesioned rats for several months and were always significantly higher than in non-lesioned controls (Fig. 3A). In the CSF, levels of AT1-AA were significantly higher in grafted rats than in non-lesioned rats and showed a non-significant tendency to be higher than in 6-OHDA lesioned rats (Fig. 3B).

**Fig. 4:**
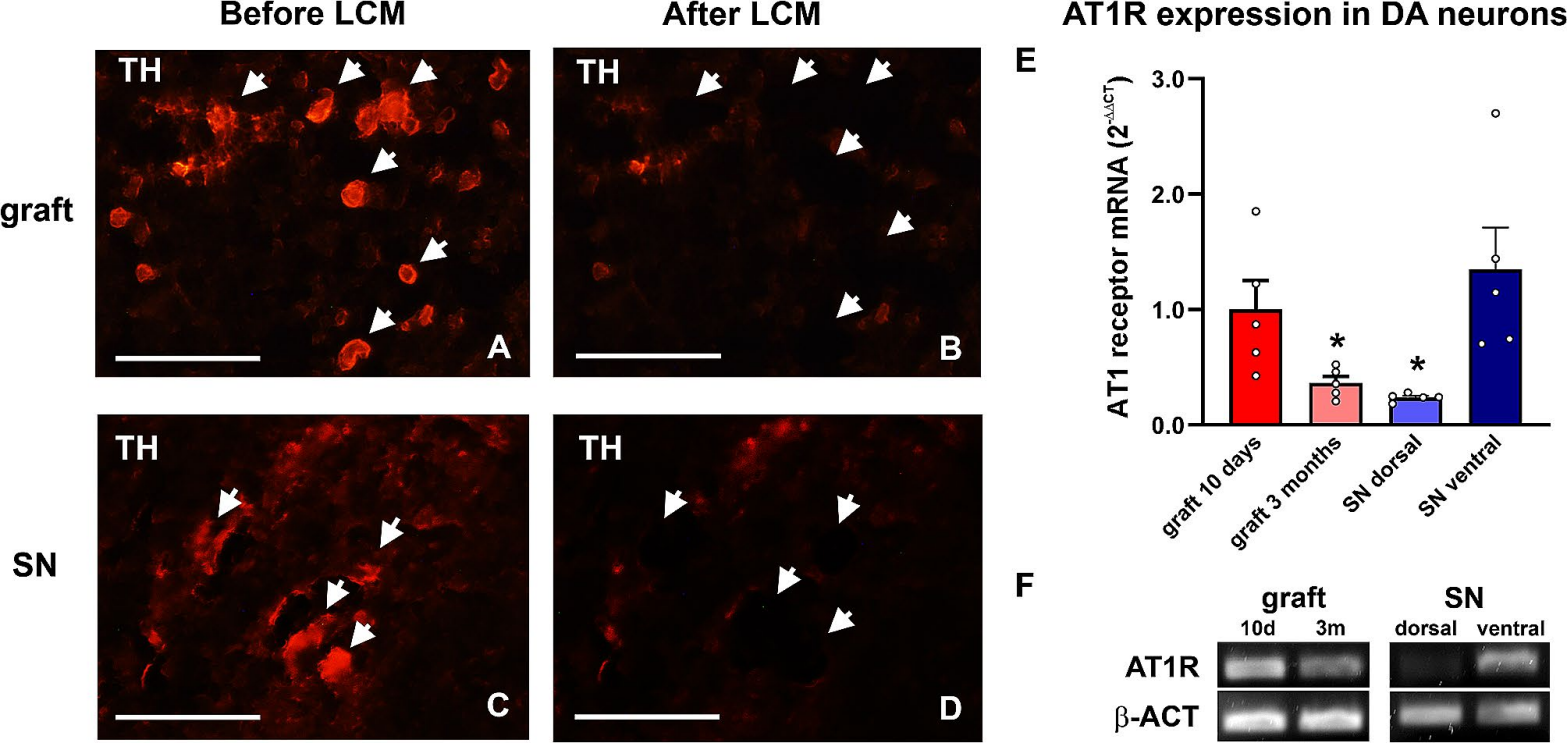
Expression of AT1 receptor mRNA in grafted dopaminergic neurons and in adult SN. Dopaminergic neurons were isolated from short- and long-term grafts and ventral and dorsal SN by LCM and detected by RT-PCR. **A-B** Photomicrographs showing grafts or **C-D** nigral sections immunolabelled for TH before and after LCM of dopaminergic neurons (arrowheads). **E** RT-PCR and **F** agarose gel analysis of AT1 receptor mRNA showed higher levels of AT1 receptor mRNA expression in dopaminergic neurons from the most vulnerable ventral SN and from 10-day-old grafts, and a significant decrease of AT1 receptor mRNA expression in dopaminergic neurons from 3-month-old grafts, which showed levels similar to those observed in less vulnerable dopaminergic neurons from the dorsal SN. The expression of the AT1 receptor gene was determined relative to the housekeeping transcripts β-actin. Full-length gels are presented in Additional file 1: Supplementary Fig. 1. Data are presented as mean ± SEM. *p < 0.05, Kruskal-Wallis One-way ANOVA on ranks followed by a Student-Newman-Keuls post-hoc test. Scale bar: 50 μm. Abbreviations: ANOVA, one-way analysis of variance; AT1, angiotensin II type 1 receptor; β-ACT, β-actin; DA, dopaminergic; LCM, laser capture microdissection; SEM, standard error of the mean; SN, substantia nigra; TH, tyrosine hydroxylase

Using LCM (Fig. 4A-F), we confirmed that grafted dopaminergic cells express mRNA for AT1 receptors at this early period and that therefore may be affected by AT1-AA activation (Fig. 4E, F). The population of dopaminergic neurons present in the grafts early after transplantation showed levels of AT1 receptor mRNA significantly higher than those present in dopaminergic neurons located in the dorsal SN of control rats and a non-significant tendency to be lower than those observed in dopaminergic neurons located in the ventral SN of control rats, suggesting the presence of both populations in the early period after grafting. Therefore, the increased levels of AT1-AA observed may act on neuronal AT1 receptors.

### Long-term effects of treatment with candesartan on survival of dopaminergic grafted precursors and microglia. Expression of neuronal AT1 receptors and AT1-AA

**Fig. 5:**
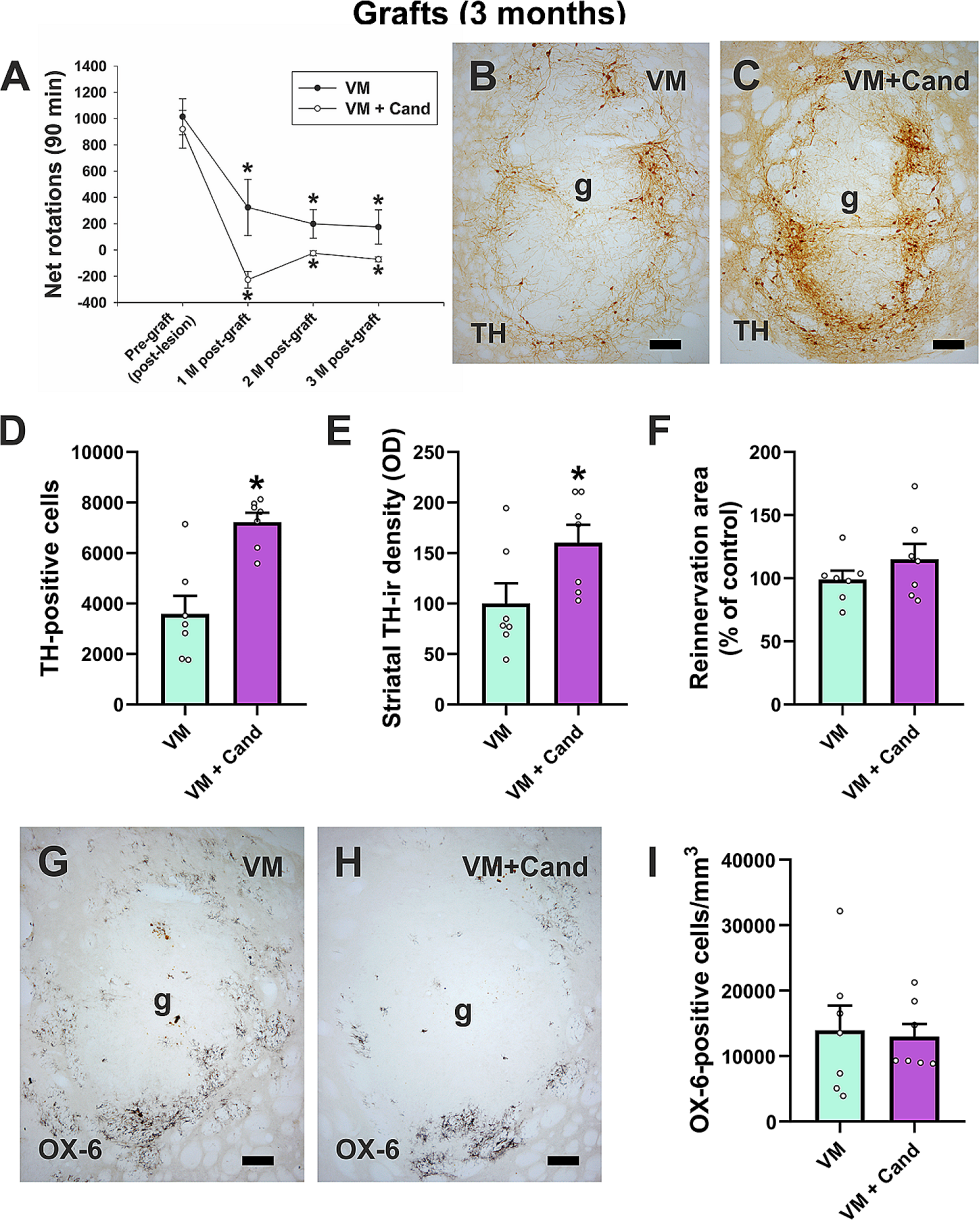
Long-term (3 months post-grafting) behavioral and histological analysis of intrastriatal grafts (g). **A** Rotational values in rats subjected to unilateral dopaminergic denervation (i.e., post-lesion and pre-grafted) and 1, 2, and 3 months after grafting in rats treated with vehicle (control group) or treated with the AT1 blocker candesartan (i.e., VM + cand). **B, C** Immunoreactivity (-ir) against TH (dopaminergic marker) in rats grafted and treated with vehicle (untreated control; **B**) or candesartan (VM + cand; **C**). **D** Graph bars showing the number of dopaminergic TH-positive neurons, **E** the density of striatal TH-immunoreactive (-ir) fibers in the reinnervation area estimated as optical density (OD) and **F** the reinnervation area (i.e., striatal host area innervated by graft-derived TH-positive fibers). **G, H** Microphotographs illustrating OX-6-positive microglial cells in rats grafted with VM cell suspensions and treated with vehicle (**G**) or candesartan (**H**). **I** Quantification of the number of OX-6 positive cells in rats grafted and treated with vehicle or candesartan. Data represent mean ± SEM. In **A**, *p < 0.05 relative to the scores obtained in dopamine-depleted rats before transplantation (i.e., post-lesion and pre-grafted) (Mann-Whitney U-test). In **D,E**, *p < 0.05 relative to control VM-grafted rats (Student’s t-test). Scale bar = 75 μm. Abbreviations: SEM, standard error of the mean; TH, tyrosine hydroxylase; VM, ventral mesencephalon

To study the effect of AT1 receptor blockade on the long-term survival of dopaminergic grafted cells, a different set of control grafts (i.e., VM-grafted) and VM + candesartan-grafted rats were sacrificed 3 months after transplantation. Before grafting, dopaminergic lesions induced a strong rotational asymmetry and amphetamine-induced ipsilateral turning (i.e., towards the denervated side). Thus, lesioned rats prepared for VM grafts showed 1014.6 ± 136.8 turns, and lesioned rats prepared for VM + candesartan showed 919 ± 143.7 turns (Fig. 5A). Amphetamine-induced rotational behavior was reverted by grafts in both groups of grafted rats (i.e., VM and VM + candesartan) 1, 2 and 3 months after transplantation compared with amphetamine-induced rotation scores before grafting (i.e., post-lesion) (Fig. 5A).

Intrastriatal grafts of fetal VM tissue contained numerous TH-positive neurons distributed in patches mainly located at the periphery of the graft (Fig. 5B). The distribution of TH-positive cells in VM + candesartan grafted animals was similar to that observed in control grafts (i.e., VM-group) (Fig. 5C). However, we observed a significantly higher number of TH-positive neurons in those animals that received VM grafts and were treated with candesartan (Fig. 5D). The average graft volume in both grafted groups (i.e., VM and VM + candesartan) was similar (0.733 ± 0.138 mm^3^ and 0.955 ± 0.051 mm^3^, respectively). The density of TH-ir fibers in the reinnervation area was higher in the rats that received VM grafts and were treated with candesartan relative to VM-grafted animals (Fig. 5E), while the extension of the reinnervation area in both grafted groups was similar (Fig. 5F). These results (i.e., a similar graft volume with increase in TH-ir neurons and fibers) suggest a decrease in selective loss of dopaminergic neurons in grafts from animals treated with candesartan.

In long-term grafts, quantitative analysis of the number of OX-6-positive cells showed that levels of microgliosis were similar in VM-grafted rats relative to rats that received VM cell suspensions and were treated with candesartan. Comparison with the number of microglial cells counted in 10-day grafts suggests that the loss of differences between groups is related to a decrease in microgliosis in the non-treated group over time (Fig. 5G-I).

Three months after grafting (Fig. 3A), serum levels of AT1-AA continued to be significantly higher than levels of healthy controls and levels of 6-OHDA-lesioned and non-grafted rats. In long-term grafts, levels of AT1-AA in CSF were significantly higher than in healthy rats, but not significantly different from non-grafted 6-OHDA-lesioned rats.

Using LCM, we confirmed that grafted dopaminergic cells express AT1 receptor mRNA in long-term grafts (Fig. 4). Interestingly, levels of AT1 receptor mRNA expression in dopaminergic neurons were significantly lower than those observed in dopaminergic neurons from grafts analyzed 10 days after transplantation, significantly lower than those observed in the ventral SN of control rats and similar to levels observed in the less vulnerable dopaminergic neurons located in the dorsal SN of control rats, suggesting that the loss of dopaminergic neurons was mostly at the expense of the most vulnerable population of the ventral SN that express high levels of AT1 receptor mRNA (Fig. 4E, F).

## Discussion

The intrastriatal grafts of mesencephalic precursors showed the usual organization, with most of the dopaminergic neurons close to the host-graft border and the TH-negative cells located in the central region of the graft. Several previous studies have described the cell composition of the TH-negative central core, which is composed of non-dopaminergic cell types such as astrocytes and GABAergic and serotonergic neurons [53, 54]. This can be minimized if a homogeneous population of stem-cell-derived dopaminergic precursors is grafted. However, recent studies suggest that a non-homogenous cell population may be more useful for graft survival [55]. Further studies using grafts of controlled compositions are needed to better understand the role of astrocytes, oligodendrocytes, and other cells for the graft vascularization, maturation, and integration of dopaminergic neurons. At ten days p.g., the number of dopaminergic neurons within the grafts was not significantly different between both groups of host animals (i.e., untreated rats and rats treated with candesartan). Rotational tests were not performed at 10 days p.g because it is known that the graft is not functional at this early period; however, there was a marked improvement in the rotational response 1 month after transplantation suggesting that the grafts had reached a threshold number of functional dopaminergic neurons and striatal reinnervation [56]. At 10 days p.g. there were significant differences between groups in the number of microglial cells infiltrating the grafts. A decrease in the microgliosis and the microglial inflammatory response after treatment with ARBs has been shown in numerous previous studies, including several in vivo and in vitro models of PD [31–33, 57, 58]. Previous studies also showed prolonged microglial activation following transplantation [59, 60], and that microglial infiltration directly correlated with the degree of rejection and worse functional outcomes [8]. In humans, surviving grafts were surrounded by activated microglia, particularly in patients who received no immunosuppression or after withdrawal of immunosuppression [9, 10] and several clinical and experimental studies have observed that increased microglial infiltration led to increased loss of grafted dopaminergic cells and increased graft-induced dyskinesias [11, 61].

We observed a significant increase in levels of circulating and CSF AT1-AA after dopaminergic lesions (6-OHDA-lesioned rats), which confirmed our recent observations in several PD models and PD patients [37, 42]. Interestingly, in the early p.g. period, there was an additional increase in circulating levels of AT1-AA (de novo formed AT1-AA), which were significantly higher than pre-grafting levels. The circulating levels significantly decreased 1-month p.g. but were still significantly higher than the pre-grafting levels (6-OHDA- lesion-induced levels, i.e., parkinsonian rat levels), and were significantly higher than levels in control rats. In the CSF, AT1-AA were significantly increased after the dopaminergic lesion and after grafting relative to healthy controls. These AT1-AA may increase AT1 receptor activity both in grafted dopaminergic neurons and host microglial cells. The pro-inflammatory and pro-oxidative effects of AT1 activation on neurons and microglial cells have been shown in many previous studies [32, 58, 62]. As previously shown in dopaminergic neurons in the nigra of adult rodents and primates, including humans [29, 34] we showed the presence of AT1 receptors in grafted dopaminergic neurons using LCM. Interestingly, levels of AT1 in 10-day grafts were similar to those observed in the most vulnerable neurons of the ventral SN, suggesting the presence of a high number of these more vulnerable neurons at this early time point, which appear to be lost in long-term grafts.

Altogether these data suggest that the presence of candesartan induces some improvement in this early period, as revealed by a decrease in the microglial infiltration and a fast recovery in the rotational behavior. However, this appears insufficient to induce significant differences in the number of surviving dopaminergic neurons, probably because other deleterious factors are predominant for dopaminergic cell death in the early p.g. period, such as lesions derived from the dissection technique, mechanical trauma, lack of growth factors/nutrients and ischemia [3, 5, 8]. Previous studies have observed AT2 receptors in dopaminergic precursors that may promote dopaminergic differentiation [63], although the expression and role of AT1 receptors in the developing brain appears much lower than those of AT2 receptors [64]. Therefore, the possibility of treatment of cell suspension with ARBs may be initially considered. However, the observed lack of significant effects on dopaminergic neuron survival during the early postgrafting period does not suggest beneficial effects of blocking AT1 receptors in the cell suspension.

At three months p.g., we observed a significantly higher number of surviving dopaminergic neurons and a higher density of striatal dopaminergic terminals in the candesartan-treated group relative to the non-treated group. Interestingly the number of surviving dopaminergic neurons in the treated group was like that observed in the 10-day-old grafts; however, there was a significant reduction in the number of dopaminergic neurons in the untreated grafts. In several human grafts, deterioration of graft function and possible loss of dopaminergic neurons was observed several months after observing graft-derived functional improvements [9, 10] which was associated with no immunosuppression or withdrawal of immunosuppression, leading to some degree of graft rejection [11]. The neuroprotection observed in the candesartan-treated group is consistent with previous studies showing that ARBs increase the survival of dopaminergic neurons subjected to different dopaminergic neurotoxins, α-synuclein overexpression, or neuroinflammation [31–33, 65]. Conversely, AT1-AA activate neuronal and glial AT1 receptors. AT1 receptors constitute the main component of the pro-oxidative/pro-inflammatory axis of the RAS (see for review [23, 24, 30]. AT1 activation leads to hydrolysis of membrane phosphatidylinositol-4,5-bisphosphate (PIP2), producing inositol trisphosphate (IP3) and diacylglycerol (DAG). IP3 activates IP3 receptors that induce the mobilization of intracellular Ca^2+^ stores [66, 67]. DAG activates protein kinase C (PKC), which activates the NADPH-oxidase complex [68, 69] the second major cell source of superoxide (i.e., behind the mitochondria) [70, 71]. NADPH-oxidase-derived superoxide and superoxide-derived reactive oxygen species are major factors in the AT1-induced pro-oxidative and pro-inflammatory effects [32, 65]. It is well known that oxidative stress, calcium dysregulation, and neuroinflammation play major roles in the pathogenesis and progression of dopaminergic neuron death [72, 73].

Interestingly, a recent study has shown that the most vulnerable dopaminergic neurons, located in the ventral tier of the human SN, which degenerate in early periods of PD, can be identified by their high expression of the AT1 receptor gene [34]. This is consistent with the higher levels of AT1 receptor mRNA observed in the dopaminergic neurons of the ventral SN in the present study and the data from LCM revealing low levels of AT1 receptor mRNA expression in the long-term surviving dopaminergic neurons, suggesting the loss of the population of dopaminergic neurons with high levels of AT1 receptor expression in the presence of high levels of AT1-AA.

After the first month, all grafted rats (i.e., VM and VM + candesartan) showed complete recovery in the amphetamine-induced rotational test relative to their pre-grafting values. This could be expected as it is well-known that above a minimum number of surviving dopaminergic cells, grafts can totally reverse rotational bias [74, 75]. Previous studies have shown that a threshold number of around 100–200 dopaminergic neurons leads to a marked reduction (> 50%) in the rotational asymmetry and that the survival of 300–500 dopaminergic neurons produced a ‘ceiling effect’ beyond which additional surviving neurons induced little or no further change in the rotational behavior [75]. While reversion of rotational asymmetry is an index of graft survival, a battery of more detailed or sophisticated behavioral tests would be necessary for detecting behavioral differences between groups of grafted rats.

Several months after grafting, we did not observe significant differences in microglial infiltration between treated and untreated rats (i.e., VM and VM + candesartan), and the comparison of the microglial counts in 10-day and long-term grafts shows that the microglial cell number was already low at 10 days in the treated group and the loss of between-group differences is related to the decrease in the number of microglial cells over time in chronic untreated grafts. Some degree of microglial infiltration was observed in chronic grafts in both groups of rats, which is consistent with previous studies showing that a low level of microglial activation is normally seen in ‘healthy’ appearing functional grafts [76] and that the persisting microglia may be indirect evidence for the synaptic integration of grafted tissue [77].

In grafted rats, we observed for several months the presence of levels of AT1-AA higher than in the PD model (6-OHDA-induced dopaminergic denervation), and higher than in healthy control rats. This is particularly interesting because AT1-AA, which act as agonists/activators of AT1 receptors, have been the non-HLA antibodies most widely associated with graft dysfunction or rejection after transplantation of different solid organs and hematopoietic stem cells [14, 15, 17–21] so that the need for AT1-AA monitoring before organ transplantation has been suggested [78]. In previous studies, we have shown that levels of serum and CSF AT1-AA are significantly increased in PD patients relative to non-PD controls [37]. In several* in vivo* and in vitro PD models we confirmed that the increase in AT1-AA in serum and CSF was related to the degeneration of dopaminergic neurons and the accompanying neuroinflammatory process [37, 42]. The mechanisms of AT1-AA production remain to be fully clarified. However, infusions of pro-inflammatory cytokines increased the serum levels of AT1-AA in animal models, and it was suggested that several cytokines induce the expression of the *Tgm2* gene (which encodes tissue transglutaminase 2, TG2), and the enzyme TG2 mediates AT1-AA generation, as it is blocked by TG2 inhibitors [79–81]. TG2 induces a posttranslational modification of the AT1 receptor at the level of its second extracellular loop, thus generating a neoantigen, which triggers the autoimmune production of AT1-AA. Furthermore, this change also induces the stabilization of the AT1 receptor as it inhibits the AT1 internalization and elimination, leading to the upregulation of surface AT1 expression and the AT1 receptor effects, particularly in the presence of increased levels of AT1-AA that activate the AT1 receptors [82–85]. Furthermore, AT1 internalization also induces intraneuronal protective responses [86, 87]. The cytokine Tumor necrosis factor superfamily member 14 (TNFSF14, also called LIGHT) appears particularly related to TG2 production [82, 83, 85, 88]. In a previous study, we observed the presence of AT1-AA in serum and CSF of 6-OHDA-lesioned rats, as in the present experiments, together with increased levels of LIGHT and transglutaminase activity in the nigral area [37]. Interestingly *TNFSF14 (LIGHT)* gene was observed to be upregulated in PD patients relative to controls and was suggested for inclusion in a set of potential gene biomarkers of PD [89].

Recent studies have shown that several brain diseases are associated with pathogenic autoantibodies that act on neuron and glial surface proteins and that the CSF from those patients has autoantigen-specific B cells that produce intrathecal synthesis of autoantibodies [90, 91]. Consistent with that observed in other brain diseases, we suggest that the dopaminergic degeneration and the accompanying neuroinflammation produce, by the above-mentioned mechanisms, an increase in modified AT1 receptors (neoantigens), which after neuron degeneration, reach the CSF, and, via the brain lymphatic system, the cervical lymph nodes. In the cervical nodes, antigen-specific B cells and circulating autoantibodies are generated. Activated B cells and/or autoantibodies may then migrate into the intrathecal compartment through the BBB, which may be facilitated because circulating AT1-AA, acting on endothelial AT1 receptors, induce BBB disruption (see below, and [37] for details). The grafting procedure induces an additional process of neuroinflammation and massive death of neurons that induce the additional increase in AT1-AA and a progressive loss of surviving dopaminergic neurons observed in the present study.

In peripheral organ transplantation, the pathogenesis of AT1-AA-mediated graft injury has been considered complement-independent, and organ cell damage, increased interstitial inflammation, microcirculation inflammation, coagulation dysregulations, and anti-angiogenic effects have been suggested as possible involved mechanisms, based on the effects of AT1 overactivation observed in previous studies in different tissues [14, 15, 17]. In recent studies, we have used in vivo and in vitro PD models to clarify the effects of AT1-AA [37, 42]. In mesencephalic neuron-glia primary cultures treated with AT1-AA, we confirmed that AT1-AA significantly increased the dopaminergic neuron death, which was inhibited by simultaneous administration of the ARB candesartan. Furthermore, AT1-AA administration also induced a high increase in tumor necrosis factor-alpha (TNFα) levels in primary cultures, possibly related to the exacerbation of the microglial pro-inflammatory response induced by AT1 activation [57] which was inhibited by candesartan. This is consistent with the present observations in grafts. However, in the case of mesencephalic grafts, the effects of AT1-AA and candesartan on graft micro-vascularization and angiogenesis may also play a relevant role by acting on AT1 receptors of endothelial cells. Furthermore, administration of AT1-AA in rats (using intraperitoneal minipumps) increased the dopaminergic cell death induced by dopaminergic neurotoxins, which was inhibited by treatment of rats with candesartan [37, 42]. High levels of circulating AT1-AA may disrupt BBB, which may also affect graft survival. We observed that administration of AT1-AA in rats (using intraperitoneal minipumps) leads to BBB disruption [37, 42] which is consistent with previous studies observing that overactivation of endothelial AT1 receptors leads to BBB disruption [92–94]. It was observed that hypertension induces BBB permeability, which is inhibited by ARBs and not by other antihypertensive drugs, which indicates that overactivation of endothelial AT1 receptors is a major factor involved in the BBB disruption and not hypertension itself [92–94].

The present study has several limitations that may be addressed in future studies. The effects of candesartan and AT1-AA observed in grafts from mesencephalic precursors remain to be confirmed for grafts of dopaminergic cells obtained from other cell sources such as ESCs and iPSCs, although similar results can be expected. We did not provide direct evidence showing that candesartan blocked the effects of AT1-AA on dopaminergic cells in the present manuscript. However, we showed this effect both in vitro and in vivo in several recent studies as detailed above [37, 42]. It is known that overactivation of neuronal AT1 receptors by AT1-AA, in dopaminergic neurons and microglial cells, promotes different mechanisms that may lead to dopaminergic neuron degeneration, as detailed above. However, these mechanisms remain to be clarified in future studies.

## Conclusion

In summary, experience with peripheral grafting reveals that, even with effective standard immunosuppression, some transplant recipients may have rejection or complications, and non-HLA antibodies, particularly AT1-AA, play a role in these cases. Standard immunosuppression primarily focuses on suppressing the immune response against HLA antigens. Even when this can induce a general immunosuppressive effect, they might not fully inhibit the production of non-HLA antibodies, which can be variable between patients. Thus, managing non-HLA antibodies usually requires a more specific and personalized approach. Techniques such as plasmapheresis and intravenous immunoglobulin therapy have been suggested. However, when upregulation of AT1-AA is observed, the use of ARBs should be considered. Candesartan has been successfully used in kidney transplantation, and the present results suggest its use in PD patients, particularly before and after dopaminergic grafts. Furthermore, the results suggest the need to monitor AT1-AA levels in PD patients, particularly in those candidates for dopaminergic grafting.

### Electronic supplementary material

Below is the link to the electronic supplementary material.

Supplementary Material 1: Additional file 1. Supplementary Fig. 1. Original uncropped gel for Fig. 4F.

Supplementary Material 2: Additional file 2. Supporting data values. Data supporting the results reported in the article.

## Data Availability

Supporting data values for all results presented in this article are available in the Additional file 2. Supporting data values. Data can also be accessed from the corresponding author, upon reasonable request.

## Abbreviations

6-OHDA: 6-hydroxydopamine
Ang II: Angiotensin II
ANOVA: One-way analysis of variance
ARBs: AT1 receptor blockers
AT1-AA: Angiotensin II type 1 receptor autoantibodies
BBB: Blood-brain barrier
cDNA: Complementary DNA
CSF: Cerebrospinal fluid
Ct: Cycle threshold
DA: Dopaminergic
DAB: 3,3´diaminobenzidine
DAG: Diacylglycerol
ELISA: Enzyme-linked immunosorbent assay
ESCs: Embryonic stem cells
IHC: Immunohistochemistry
IP3: Inositol trisphosphate
iPSCs: Induced pluripotent stem cells
-ir: Immunoreactive
KPBS-BSA: Potassium phosphate-buffered saline containing 1% bovine serum albumin bovine serum albumin
LCM: Laser capture microdissection
m: Months
M-MLV: Moloney murine leukaemia virus
mRNA: messenger RNA
NA: Numerical Aperture
p.g.: Post-grafting
p.l.: Post-lesion
PD: Parkinson´s disease
PKC: Protein kinase C
RLU: Relative luminescence units
RT: Reversed transcribed
SEM: Standard error of the mean
SN: Substantia nigra
TH: Tyrosine hydroxylase
TNFSF14: Tumor necrosis factor superfamily member 14
TNFα: Tumor necrosis factor-alpha
VM: Ventral mesencephalon
