# Supplementary figures and images for: Non-HLA angiotensin-type-1 receptor autoantibodies mediate the long-term loss of grafted neurons in Parkinson’s disease models
Source: Stem Cell Res Ther. 2024 May 12;15:138. doi: 10.1186/s13287-024-03751-y (PMC11089721; doi:10.1186/s13287-024-03751-y)

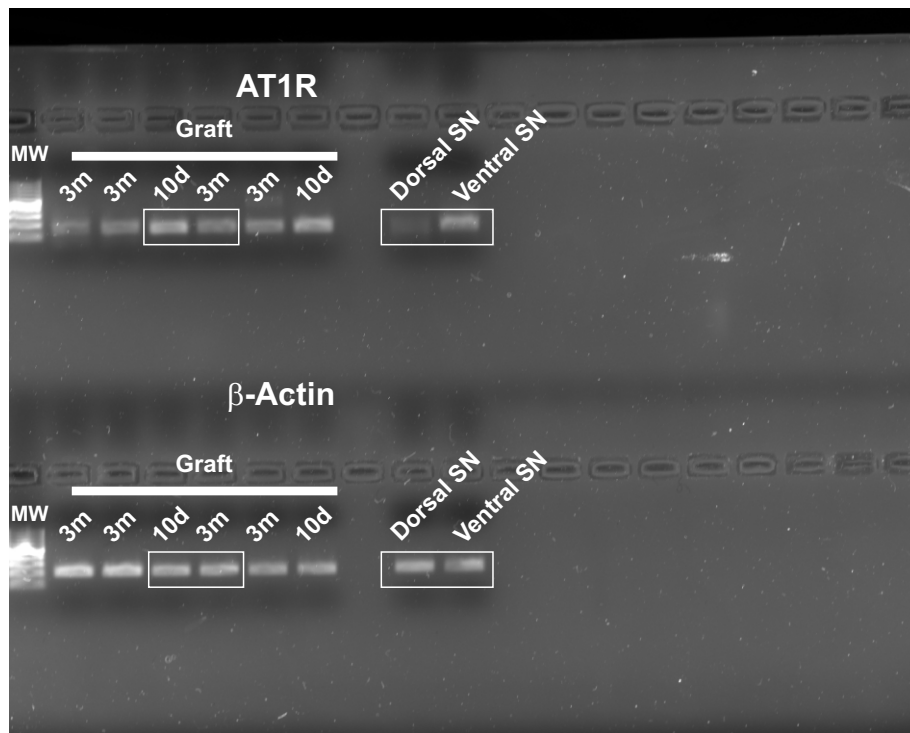

**Supplementary Fig. 1.** Original uncropped gel for Fig. 4 F

Supplement: Supplementary file 1 — Supplementary Material 1: Additional file 1. Supplementary Fig. 1. Original uncropped gel for Fig. 4F. [file 13287_2024_3751_MOESM1_ESM.pdf]
